# How Framed Messages Influence Depression Assessment Intentions: Interactivity of Social Media as a Moderator

**DOI:** 10.3390/ijerph18041787

**Published:** 2021-02-12

**Authors:** Quan Gao, Hye Eun Lee

**Affiliations:** School of Communication and Media, Ewha Womans University, Seoul 03760, Korea; cenniee.ko@gmail.com

**Keywords:** depression, message framing, interactivity, social media, intention to take an assessment

## Abstract

This study examines how the framing and interactivity of messages influence the intentions of individuals to take a depression assessment. An experiment with a 2 (message framing: gain-versus loss-) × 2 (interactivity: low versus high) between-subject design was conducted among 269 Chinese participants (*M* = 30.70, *SD* = 7.34). The results showed that those reading loss-framed messages had a higher intention to take a depression assessment compared to those reading gain-framed messages. Secondly, those reading messages delivered with high interactivity had a higher intention to take a depression assessment than those reading messages delivered with low interactivity. Further, the interaction effect of framed messages and their varying degrees of interactivity was found to influence the intentions of individuals to take a depression assessment as well. Specifically, participants who read the loss-framed message reported stronger intention in the high interactivity group. In contrast, there was no significant difference between the effectiveness of loss-framed and gain-framed messages in promoting the intention to take a depression assessment in the low interactivity condition.

## 1. Introduction

WHO (2017) found that at least 300 million people (4.4% of the global population) suffer from depression [1]. Depression patients experience the loss of pleasure and interest, changes in sleep and appetite, feelings of sorrow and guilt, and low energy and low self-esteem [2]. Furthermore, depression is a significant contributing factor to suicide. Around 800,000 people died of suicide in 2015, taking up 1.5% of all deaths globally and functioning as the 20th leading cause of death [1]. In China, about 200,000 patients with depression commit suicide every year [3]. The latest China Mental Health Survey (CMHS) in 2019 demonstrates that the lifetime and 12-month prevalence of the depressive disorder in China are 6.8% and 3.6% [4]. Despite the prevalence and severity of depression in China, the screening rate of depression is under 10% of people with depression due to social discrimination and incorrect knowledge of depression [5]. Furthermore, research demonstrates that 70.6% and 56.1% of the major depressive disorder patients experienced clinical and functional remission after therapy [6]. Thus, taking a depression assessment and getting treated for depression as early as possible is essential.

With this increasing prevalence of depression, social media usage has become more heavily scrutinized. Studies have mostly investigated depression phenomena through the lens of social media, ranging from conducting depression assessments through posts on social media [7,8] to examining the correlation between social media usage and depression [9,10]. However, only a few studies have investigated how social media influences the intentions of individuals to take a depression screening. Furthermore, even in the research on other media, scholarly consensus has not been reached on the persuasiveness of messages on the promotion of depression assessment behaviors [11,12,13]. Therefore, this research studies (1) how differently framed messages affect the intentions of individuals to take a depression assessment (heretofore, depression assessment intentions), and (2) whether social media moderates the effectiveness of framed messages to promote depression assessment intentions.

Messages swaying the behavior of individuals toward disease detection can be framed in many ways, including in terms of gain versus loss. According to prospect theory, gain-framed messages imply associated benefits, whereas loss-framed messages suggest possible costs [14]. Consider the statement “We should not smoke for the sake of our health”. This claim can be construed in the form of a gain-framed message such as, “If we start to give up smoking, it will be possible to keep your lungs healthy”. It can also take the form of a loss-framed message such as, “If we continue smoking, it will be possible for us to suffer from lung cancer”. The varying effects of gain-framed versus loss-framed messages have been tracked in numerous studies, ranging from breast self-examination (BSE) research [15] and human immunodeficiency virus (HIV) test promotion among women [16] to alcohol intake reduction among psoriasis patients [17].

Tversky and Kahneman (1981) pose the prospect theory to explain how people make choices when encountering gains and losses [18]. Participants were asked to choose between two options in the event that a disease were to break out: (1) if they chose option A, then 200 people would be saved, and (2) if they selected option B, then there would be a one-third probability that 600 people would be saved and a two-thirds probability that 400 people would die. In this situation where possible gains were emphasized, participants were more likely to choose option A, implying that participants gravitated toward less risk. In the other situation where possible losses were emphasized, participants were asked to select from the following two options: (1) if they chose option C, then 400 people would die, and (2) if they chose option D, then there was a one-third probability that no one would die and a two-thirds probability that 600 people would die. Participants in this group tended to choose option D, which contains more risk. The study concludes that, even if the expected outcomes are identical, individuals tend to avoid taking a risk when the message focuses on potential gains. Individuals are likely to choose the sure gains rather than the possible larger gains with larger uncertainty. On the contrary, individuals prefer taking a risk when the message emphasizes possible losses. That is, when faced with possible losses, individuals start to take the gamble in the hope of paying nothing. Table 1 explains the two hypothetical situations of the prospect theory.

Loss-framed messages are more effective than gain-framed messages in promoting disease detection behaviors because those behaviors contain a higher degree of risk [19,20]. Disease detection promotion messages encompass two kinds of risk: (1) the short-term risk that a disease will be detected, and (2) the long-term risk that once detected, the disease cannot be treated and thus will have an adverse effect on the patient’s health. Prospect theory postulates that most people are risk-averse when considering benefits but risk-seeking when focusing on costs. [18]. When messages are framed in a gain way (i.e., if you take BSE, you will have a healthy breast), without the long-term risk that the disease cannot be treated, individuals tend to avoid an examination that may result in the detection of disease. On the contrary, loss-framed messages (i.e., if you do not take BSE, it is possible that you may suffer from breast cancer), can make long-term risk salient. Thus, individuals tend to take the depression assessment so that they maximize their chance of avoiding the long-term risk.

There is much research indicating that loss-framed messages encourage stronger behavioral intentions than gain-framed messages. For instance, messages framed in a loss way were found to be more effective when utilized to promote the examination of coronary heart disease to undergraduates [21], HIV tests to women [22], a mammogram to women [19], and self-examination of skin cancer to undergraduates [23]. Regarding depression-related messages in particular, although some researchers have found that patients with depression pay more attention to loss-framed messages [11], other researchers have found that gain-framed reminder letters encouraged individuals to attend their medical appointments to a greater degree than neutral reminder letters. Additionally, there was no significant difference between the effects of loss-framed messages and gain-framed messages [12,13]. In light of the inconsistent results on whether gain-framed or loss-framed messages are more effective, this paper poses the following research question:

**RQ1:** Will individuals have different intentions to take a depression assessment after reading gain-framed messages versus loss-framed messages?

Not only the framing of messages, but also interactivity, would influence the intentions of individuals to take a depression assessment. Researchers define interactivity as modification of content [24,25], instant responsiveness [26], user intention fulfillment [27], perceived interpersonal communication [28], technological affordances [29], and reciprocal communication or synchronicity [30]. Moreover, Sundar (2007) also tried to conceptualize interactivity into one model, concluding that there are three forms of interactivity: modality (medium) feature, message feature, and source feature [29]. Modality interactivity refers to the tools or features available on the interface that make users interact with information such as sliders and drags [31]. Message interactivity is the degree to which communication between users and the system can be realized within a system (i.e., hyperlinks) [32]. Source interactivity refers to the user’s capability to manipulate the source of messages [29]. Actions such as liking, commenting, and sharing content are examples of source interactivity [33].

This research deems the interactivity of social media as source interactivity because when compared to other websites, the affordances of social media (i.e., liking, commenting, and sharing) make individuals feel they may be the source of the content [34]. Source interactivity can provide a greater sense of agency, which leads to more user engagement [33]. When individuals like, comment, or share a message on social media, express their opinions and attitudes, and perform their duties as a gatekeeper, many cognitive resources are triggered to process the message. Further, systematic processing entails much more cognitive resources compared to heuristic processing [35]. Thus, it can be inferred that systematic processing may be elicited during this process.

How the information is processed will influence an individual’s behavior [36]. For instance, Hovick et al. (2011) studied how individuals living at or near the poverty line process risk information, and the results demonstrated that health-protective behaviors can be boosted by systematic processing [37]. Additionally, in the study on how messages influence customers’ behaviors during a product-harm crisis, the results showed that systematic processing can influence individuals’ intention to behave in a certain manner [38]. As mentioned above, source interactivity can contribute to systematic processing, which can increase behavior responses. Thus, the following hypothesis is proposed:

**Hypothesis** **1** **(H1):** *Individuals who read messages with high interactivity will have a higher intention to take a depression assessment than those who read messages with low interactivity*.

Moreover, interactivity will contribute to increased systematic processing [31]. The heuristic systematic model (HSM) indicates two processing methods: heuristic and systematic processing [35]. In the systematic process, recipients actively elaborate on persuasive messages. On the other hand, in heuristic processing, people process information through simple decision rules, or heuristics, such as accessibility and applicability. When systematic processing is elicited, individuals tend to deliberately evaluate a message’s diagnosticity based on their prior knowledge [21]. Messages with a higher perceived diagnosticity are more effective messages. Loss-framed messages, or negative information, have been found to be more informative and diagnostic [39]. Therefore, loss-framed messages are more persuasive. Empirical research also demonstrates that negatively framed messages are more effective than positively framed messages due to greater perceived diagnosticity [40]. Thus, the following hypothesis is posited:

**Hypothesis** **2** **(H2):** *When messages are delivered with high interactivity, individuals who read loss-framed messages will have a higher intention to take a depression assessment than those who read gain-framed messages*.

## 2. Materials and Methods

### 2.1. Design and Participants

This research conducted a 2 (message framing: gain- versus loss-) × 2 (interactivity: low versus high) between-subject experiment to test the hypotheses. The experiment was implemented through an online survey that was created in English and then translated into Chinese. To ensure the equivalencies of the survey items, the researchers followed translation, back-translation, and review by other experienced researchers. A pretest was utilized to assess the initial intention to take a depression assessment, the involvement of pre-existing issues (i.e., how a participant feels about depression), depression level, and perceived risk. During the post-test, researchers measured the perceived message framing, perceived interactivity, and the intention to take a depression assessment. The study has been approved by the ethical committee of Ewha Womans University in South Korea.

269 Chinese (147 females) were recruited through a survey company “Wenjuanxing” in China, and those who are at least 18 years old and able to use social media could participate in the study. Three participants were ruled out in the final analyses due to incomplete surveys. The average age of participants was 30.70 (*SD* = 7.34, *Range* = 18–63), and they reported using social media for an average of 3.79 h per day (*SD* = 2.28, *Range* = 0.50–15.00). Table 2 provides the characteristics of the study participants.

This experiment was conducted in the form of an online survey. First, subjects were asked to take a survey with an informed consent form, which asked them to report their gender, age, social media usage, issue involvement, perceived risk of depression, depression level, and the initial intention to take a depression assessment. Secondly, participants were randomly assigned to four conditions: (1) the gain-framed message delivered with high interactivity, (2) the gain-framed message delivered with low interactivity, (3) the loss-framed message delivered with high interactivity, and (4) the loss-framed message delivered with low interactivity. There were different messages on the stimulated websites persuading participants to take a depression assessment. Participants were then asked to read the stimulus and to complete the survey measuring their perceived message framing, perceived interactivity, and intention to take a depression assessment.

### 2.2. Stimulus and Manipulation

Two kinds of messages (gain-framed and loss-framed) were presented separately in stimulated social networking sites of high interactivity and low interactivity. The content of the message was the mixed version of the message utilized by Hull (2012) [16] and Lueck (2017) [11]. Gain-framed stimuli focused on the benefits of depression assessment: “If you take a depression assessment, you will gain emotional support, mental well-being, less stress, energy, concentration, sleep, and the interest in hobbies and daily activities will last.” On the contrary, loss-framed stimuli illustrated the loss of not taking depression detection: “If you do not take a depression assessment, you will lose out on emotional support, mental well-being, less stress, energy, concentration, sleep. Also, the loss of interest in hobbies and daily activities will last.”

Interactivity was manipulated as the degree to which subjects can interact with the websites and the messages. We manipulated the high interactivity site as a Q&A social network site. The participants were provided with stimuli through Zhihu, a famous Q&A social network site in China, which allowed them to reply, and to like, or share the stimuli. Moreover, the message of low interactivity was offered through a mock wiki website. Participants could only read the stimuli and could not reply to, like, or share the stimuli.

An independent small-sample t-test (N = 40) before the experiment was implemented to independently assess participants’ perceived message framing and perceived interactivity. As expected, participants who were instructed to read the gain-framed message perceived that the stimulus assigned to them was gain-framed to a greater degree (*M* = 4.05, *SE* = 0.11) than those instructed to read the loss-framed message (*M* = 2.95, *SE* = 0.18), *t*(74) = 5.27, *p* < 0.001. Moreover, participants assigned to the loss-framed message group reported that the stimulus was loss-framed to a greater degree (*M* = 3.76, *SE* = 0.13) than those in the gain-framed message group (*M* = 2.42, *SE* = 0.14), *t*(74) = 7.11, *p* < 0.001. For perceived interactivity, the high interactivity group reported that the website was more interactive (*M* = 3.97, *SE* = 0.09), whereas the low interactivity group reported less interactivity (*M* = 2.30, *SE* = 0.16), *t*(70) = 9.33, *p* < 0.05.

### 2.3. Measures

#### 2.3.1. Manipulation Check

*Perceived Message Framing.* Subjects were asked to indicate whether the stimulus assigned to them is gain-framed or loss-framed. A 5-point Likert Scale was utilized. Their perceptions were assessed by the modified statements used in previous research [11]. The extent to which a message is gain-framed was measured by the item “The arguments in the message focus on the benefits of seeking help” (*M* = 3.68, *SD* = 0.94), whereas the degree of loss-framing was assessed by “The arguments in the message focus on the costs of not seeking help” (*M* = 3.28, *SD* = 1.02).

*Perceived Interactivity.* Three modified items, including “The website is interactive”, “The website allows me to perform a lot of actions”, and “The website allows me to access information in a variety of ways” [41] were measured by a 5-point scale. (*M* = 3.76, *SD* = 0.75, *Cronbach’s α* = 0.78).

#### 2.3.2. Dependent Variable

*The Intention to Take a Depression Assessment.* The intention to take a depression assessment was measured by a 5-point scale (1 = “*strongly disagree*”; 5 = “*strongly agree*”) adapted from the items measuring the behavioral intentions to take an HIV test [16]. The items are “I will take an assessment for depression”, “I plan to take an assessment for depression”, and “I intend to take an assessment for depression” (*M* = 2.72, *SD* = 1.00, *Cronbach’s α* = 0.87).

#### 2.3.3. Control Variables

*Demographic factors.* The gender, age, and average daily time on social media usage for each participant were controlled.

*The Initial Intention to Take a Depression Assessment.* The initial intention to take a depression assessment was measured by the same 5-point scale (*M* = 2.30, *SD* = 0.99, *Cronbach’s α* = 0.88).

*Issue Involvement.* Issue involvement was controlled, as it was indicated to influence how framed messages influence the intention to take an assessment [42]. It was measured by adapted items through which subjects evaluated information about depression [43]. The example adjectives are “unimportant—important”, “of no concern—of concern to me”, “irrelevant—relevant”. Higher scores represent higher issue involvement (*M* = 4.57, *SD* = 1.24, *Cronbach’s α* = 0.91).

*Perceived Risk.* Perceived risk refers to the extent to which individuals can feel the negative test result [16]. Because perceived risk was found to moderate how framed messages influence disease detection behaviors [44], it was controlled in this research. Perceived risk was assessed using a two-item scale adapted from Hull’s research [16]. The items are as follows: “How likely is it that you have depression?” and “How likely is it that you will get depression in the future?”. “Extremely unlikely” responses were considered “very low perceived risk”, whereas “extremely likely” represented “very high perceived risk” (*M* = 2.54, *SD* = 1.00, *Cronbach’s α* = 0.84).

*Depression.* Pessimism, an indicator of depression, can affect the process by which framed messages promote detection behavior [14]. Therefore, depression was controlled. It was measured by a 5-point scale modified from The Burns Depression Checklist [45]. Example items include “feel sad or down in the dumps”, “Loss of motivation”, and “Difficulty sleeping or sleeping too much” (*M* = 2.14, *SD* = 0.69, *Cronbach’s α* = 0.94). Table 3 shows the key measures.

## 3. Results

### 3.1. Statistical Analysis

A general linear model (GLM) was used to test hypotheses using SPSS Statistics 25.0 (IBM, Chicago, IL, USA). The two types of messages (gain-framed versus loss-framed) and interactivity (low versus high) were independent variables, and the intention to take a depression assessment was a dependent variable in the model. The gender, age, daily social media usage, the initial intention to take a depression assessment, issue involvement, depression, and perceived risk of the participants were controlled. Table 4 demonstrates the descriptive analyses and correlations among control and dependent variables.

### 3.2. The Main Effect of Message Framing on the Intention to Take a Depression Assessment (RQ1)

**Figure 1:**
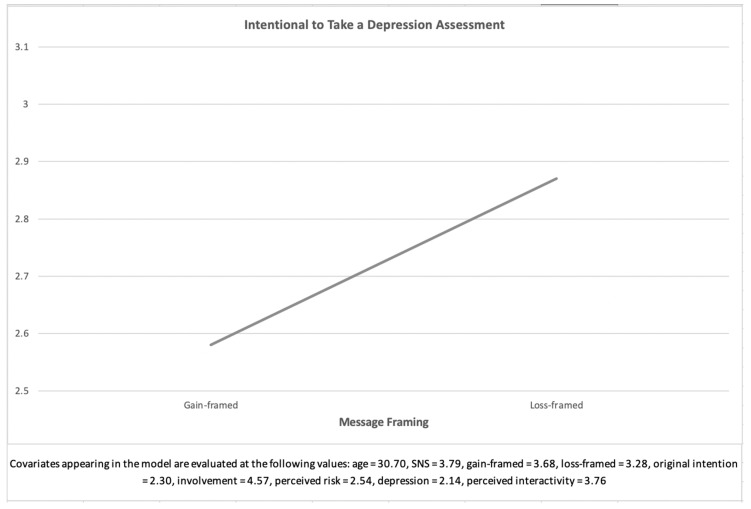
Main effect of framed messages on the intention to take a depression assessment (RQ1).

The analysis showed that the main effect of message framing on the intention to take a depression assessment was significant, *F*(1,258) = 17.41, *p* < 0.001, *η*^2^ = 0.06. Specifically, after reading the manipulated message, participants who were requested to read the loss-framed message were more willing to take a depression assessment (*M* = 2.91, *SE* = 0.09) than those requested to read the gain-framed message (*M* = 2.55, *SE* = 0.08). Thus, the research question was answered as follows: loss-framed messages elicited the intention to take a depression assessment to a greater degree than gain-framed messages. Figure 1 shows the main effect of framed messages on the intention to take a depression assessment.

### 3.3. The Main Effect of Interactivity on the Intention to Take a Depression Assessment (H1)

**Figure 2:**
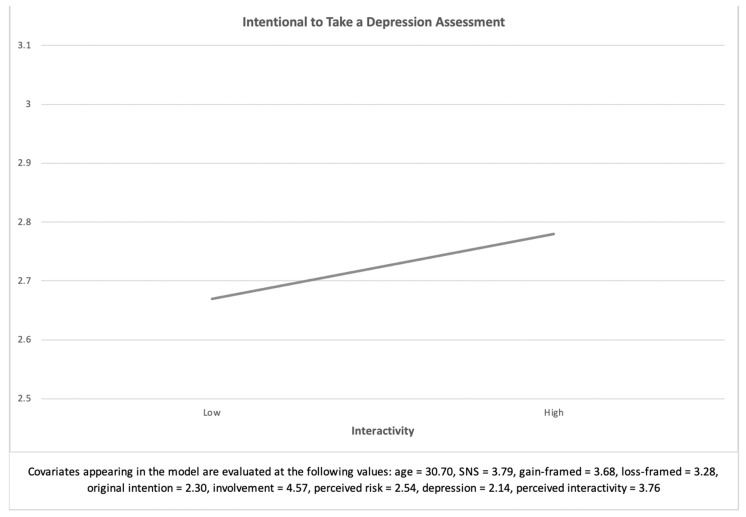
Main effect of interactivity on the intention to take a depression examination (H1).

The analysis demonstrated that the main effect of website interactivity on the intention to take a depression assessment was significant, *F* (1, 258) = 4.23, *p* < 0.05, *η^2^* = 0.02. Participants reading the message with high interactivity (*M* = 2.78, *SE* = 0.08) had a stronger intention to take a depression assessment than those reading the message with low interactivity (*M* = 2.66, *SE* = 0.09). Therefore, H1 was supported. Figure 2 shows the main effect of interactivity on the intention to take a depression assessment.

### 3.4. The Interaction Effect of Message Framing and Interactivity on the Intention to Take a Depression Assessment (H2)

**Figure 3:**
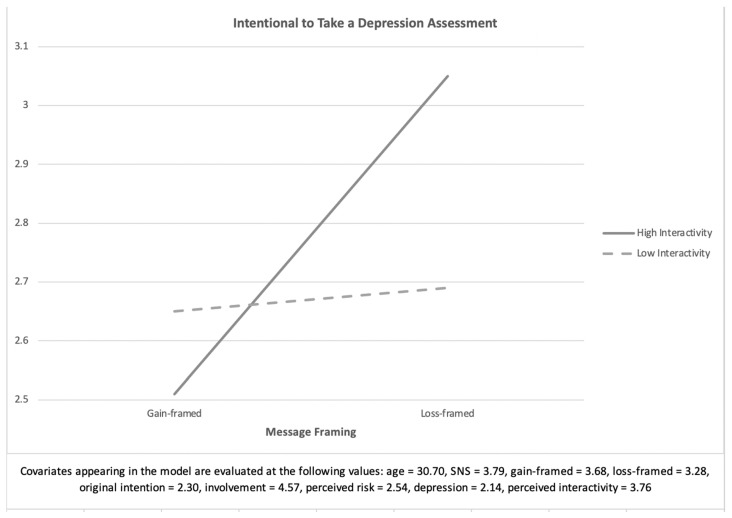
Interaction effect of framed messages and interactivity of social media (H2).

The analysis was used to examine whether there exists an interaction effect of message framing and website interactivity on the intention to take a depression assessment. The result revealed that the interaction effect was significant, *F* (1, 258) = 11.53, *p* < 0.05, *η^2^* = 0.04. Scheffe’s post hoc analysis showed that the difference between the loss-framed message delivered with high interactivity (*M* = 3.16, *SE* = 0.17) and the gain-framed message delivered with high interactivity (*M* = 2.42, *SE* = 0.17) was significant at *p* < 0.001, whereas low interactivity was not significant. As Figure 3 shows, those who were instructed the loss-framed message had a stronger intention to take a depression assessment than those who were instructed to read the gain-framed message when messages were delivered through a social network website with high interactivity. Hence, H2 was supported. Figure 3 shows the interaction effect of framed messages and interactivity on the intention to take a depression assessment.

## 4. Discussion

Message framing has been indicated to influence individual health behaviors, such as detection, prevention, or recovery from diseases [14]. For detection behaviors, loss-framed messages have been found to enhance the intention to take an assessment to a greater degree than gain-framed messages [23]. When individuals are faced with loss-framed messages focusing on associated costs, they tend to be risk-liking [18]. Moreover, to take a depression assessment implies there is a possibility that a disease may be detected [7]. Thus, individuals are more prone to taking a depression assessment when they read loss-framed messages.

This research investigated how framed messages influence the intention to take a depression assessment in response to the inconsistent research results of the effects of message framing on the intention to take a depression assessment [11,12,13]. Unlike the research of Mavandadi et al. (2018), that demonstrated that gain-framed messages encourage the appointment attendance of depression patients to a greater degree [13], this study found that loss-framed messages elicit stronger intention to take a depression assessment. The possible explanations are as follows. First, Mavandadi et al. (2018) focus on patients with depression, whereas this study was implemented among undiagnosed people with controlled depression levels. Research shows that patients with depression are in a negative cognitive state, which biases their information processing negatively [46]. The loss-framed messages may fail to elicit intention to perform certain behaviors because they are accustomed to the negative cognitive and emotional responses entailed by various stimuli. Moreover, the Asian participants in the research of Mavandadi et al. (2018) only take up 1% (N = 348) of the sample. In contrast, the participants in this study are all Chinese. Research indicates that loss-framed messages impact the intention to floss among East Asian participants more strongly than gain-framed messages, whereas gain-framed messages are more effective on their Caucasian counterparts [47]. However, flossing is a prevention behavior, and gain-framed messages have been found to be more effective in promoting prevention behaviors [14]. The reverse effect may be attributed to the cultural characteristics of East Asians and can also occur in detection behaviors. We suggest future research to explore message framing’s effects on detection behaviors based on differences in ethnicity and culture.

Secondly, this study attempted to examine whether the interactivity of social media would influence the intention to take a depression assessment, as the prior studies demonstrated that higher website interactivity had stronger effectiveness, such as more absorbed cognition, more positive attitudes, stronger enjoyment, and increased behavioral intentions [34]. The results demonstrated that the main effect of interactivity on the intention to take a depression assessment was significant. That is, individuals could experience significantly different intentions to take an assessment depending on whether they approached depression assessment promotion messages with high interactivity or low interactivity. Specifically, they were more motivated to take a depression assessment when promotion messages were delivered with high interactivity than with low interactivity. This can be explained by the importance of source interactivity over modality interactivity and message interactivity.

Interactivity consists of modality interactivity, message interactivity, and source interactivity [29]. Prior studies mainly focused on the domain of modality interactivity and message interactivity, and it was indicated that modality interactivity was more effective on cognition, attitudes, enjoyment, and behavioral intentions compared to source interactivity and message interactivity [34]. However, the current study found that source interactivity can also exert a significant influence on the persuasiveness of messages. Research indicated that the functions of social media, such as liking, commenting, and sharing, belong to source interactivity because they can make users feel a greater sense of agency [33]. As discussed above, when individuals like, comment, or share a message, they will feel more immersed in the experience than if they were to simply read a message. This kind of immersion is identified as “intense concentration or flow”, referring to the perceived control and challenges when users approach computers [48]. In this process, many cognitive resources are utilized when individuals like, comment, or share a message, which may elicit systematic processing [28]. Further, some research demonstrated that systematic processing can be a contributor to behavioral intention change [37,38]. Another explanation may be that the functions of social media, such as sharing, will enhance users’ engagement by strengthening their identification and connection with the content [49].

Furthermore, the present research tested whether social media’s interactivity moderated the relationship between message framing and the intention to take a depression assessment. As hypothesized, interactivity did serve the moderating role, implying that the effect of message framing on depression assessment promotion was only significant for the group with high interactivity. One explanation is that, when systematic processing is induced by high interactivity [31], individuals evaluate the message based on deliberation and the perceived evaluation of the messages [40]. Loss-framed messages have been found to be more diagnostic and informative than gain-framed messages [41]. Hence, loss-framed messages should entail more persuasion or more favorable judgments [42]. Another possible explanation is that individuals make judgments based on heuristic cues when the message is of low interactivity. Slovic et al. (2004) suggest that affect can be heuristic cues—people consult the affect pool for judgments [50]. If their emotion toward an activity is positive, they tend to perceive the risks as low and the benefits as high. Gain-framed messages may arouse positive emotional responses, contributing to increased perceived benefits and decreased perceived risk associated with the depression assessment. Consequently, the effect of loss-framed messages on the intention to take a depression assessment is attenuated in the low interactivity condition. Future research can investigate whether heuristic processing is elicited to a greater degree than systematic processing in the low interactivity condition and whether affect, as a heuristic cue, can mediate the relationship between message framing and behavioral intentions.

There exist several limitations to this research. First, this research asked participants to self-report their intention to take a depression assessment rather than their behaviors regarding whether they would really take a depression assessment. Furthermore, participants were asked to report their intention to take a depression assessment immediately upon reading the stimulus, leaving the long-term effect unknown. Thus, future studies are recommended to examine whether participants will take a depression assessment long after they are exposed to framed messages with different degrees of interactivity. Further, although this research hypothesized that systematic processing was triggered through message elaboration; it did not test whether message elaboration was elicited. It is recommended that future studies assess message elaboration, as it was generally manipulated as a self-reported elaboration and a thought-listing measure [31]. Self-reported elaboration can be measured by an item such as “I thought about what actions I myself might take based on what I browsed” [51]. Additionally, the thought-listing measure can be implemented by asking participants to list all the thoughts while being exposed to the websites [31]. Moreover, although the research demonstrated the main effect of message framing on depression assessment behavioral intention, it did not examine the possible mediating variables, such as the attitudes towards the messages. Future studies should try to seek possible variables mediating the effects of message framing on the intention to take a depression assessment.

## 5. Conclusions

This study yields insight into how the framing and interactivity of messages influence individuals’ intentions to take a depression assessment. The findings suggest that loss-framed messages boost the intention to take a depression assessment to a greater degree than gain-framed messages. The possible explanations are negatively biased cognition [46], and ethnic and cultural differences. In addition, messages delivered with high interactivity elicit a stronger intention to take a depression assessment than messages with low interactivity. This is because interactivity enhances cognitive absorption, which boosts systematic processing [28,48]. Finally, loss-framed messages entail a stronger intention to take a depression assessment, whereas there is no significant difference between the effectiveness of loss-framed and gain-framed messages. The two possible explanations are perceived evaluation of messages during systematic processing [40,41,42] and positivity bias during heuristic processing [50]. Our findings contribute to the message design for depression assessment promotion.

## Figures and Tables

**Table 1:** Two hypothetical situations explaining prospect theory.

	Option	
Emphasizing possible gains	A: “200 patients are sure to be saved”	B: “A 33% chance of saving all 600 patients and a 66% chance of saving no one”
Emphasizing possible loses	C: “400 patients are sure to die”	D: “A 33% chance of no patients dying and a 66% chance of all 600 patients dying”

**Table 2:** Characteristics of the study participants (*n* = 269).

	Mean (SD) or Percentage	Range
Age	*M* = 30.70 *SD* = 7.34	18–63
Gender	Male: 122 (45.0%) Female: 147 (55.0%)	
Social Media Usage (h)	*M* = 3.79 *SD* = 2.28	0.50–15.00

**Table 3:** Key measures.

Measure	Response Options	Reliability (Cronbach’s Alpha)
The initial intention to take a depression assessment	1 (strongly disagree) –5 (strongly agree)	0.88
I will take an assessment for depression. I intend to take an assessment for depression. I plan to take an assessment for depression.		
Issue involvement (How you feel about depression)	1 (close to the adjective on the left)–7 (close to the adjective on the right)	0.91
Important—unimportant * Of no concern—of concern to me Irrelevant—relevant Means a lot to me—means nothing to me * Trivial—fundamental Matters to me—doesn’t matter to me * Uninterested—interested Significant—insignificant * Vital—superfluous *		
Perceived risk	1 (extremely unlikely)–5 (extremely likely)	0.84
How likely is it that you have depression? How likely is it that you will have depression in the future?		
Perceived depression	1 (not at all)–5 (extremely)	0.94
Feeling sad or down in the dumps. Feeling hopeless Low self-esteem Feeling worthless or inadequate Criticizing yourself or blaming yourself Difficulty making decisions Loss of interest in family Loneliness Spending less time with family or friends Loss of motivation Loss of interest in work and other activities Loss of pleasure or satisfaction in life Feeling tired Difficulty sleeping or sleeping too much Decreased or increased appetite Loss of interest in sex Worrying about your health Do you have any suicidal thoughts? Would you like to end your life? Do you have a plan for harming yourself?		
The intention to take a depression assessment	1 (strongly disagree)–5 (strongly agree)	0.87
I will take an assessment for depression. I intend to take an assessment for depression. I plan to take an assessment for depression.		

Note. * Items were reverse coded.

**Table 4:** Descriptive analysis and correlations between control and dependent variables.

	1	2	3	4	5	6	7
1 Age	-						
2 SNS use	−0.22 **	-					
3 Initial intention to take depression assessment	−0.01	−0.69	-				
4 Issue involvement	−0.16 **	−0.01	0.42 **	-			
5 Perceived risk	−0.06 **	0.06	0.70 **	0.46 **	-		
6 Perceived depression	−0.10	0.04	0.65 **	0.33 **	0.70 **	-	
7 The Intention to take depression assessment	−0.10	0.33	0.74 **	0.52 **	0.65 **	0.55 **	-
Mean	30.70	3.79	2.30	4.57	2.54	2.14	2.72
Standard deviation	7.34	2.28	0.99	1.24	2.00	0.69	1.00

Note. *n* = 269. ** *p* < 0.01.

## Data Availability

Qualified researchers can obtain the data from the corresponding author (hyeeunlee77@ewha.ac.kr). The data are not publicly available due to privacy concerns imposed by the IRB.
